# Replicating phages in the epidermal mucosa of the eel (*Anguilla anguilla*)

**DOI:** 10.3389/fmicb.2015.00003

**Published:** 2015-01-29

**Authors:** Miguel Carda-Diéguez, Carolina Megumi Mizuno, Rohit Ghai, Francisco Rodriguez-Valera, Carmen Amaro

**Affiliations:** ^1^ERI Biotecmed, University of Valencia, ValenciaSpain; ^2^Evolutionary Genomics Group, Department de Producción Vegetal y Microbiología, Universidad Miguel Hernández, San Juan de AlicanteSpain

**Keywords:** metagenomics, phage, eel, mucosa, immunity

## Abstract

In this work, we used the eel (*Anguilla anguilla*) as an animal model to test the hypothesis of Barr et al. (2013a,b) about the putative role of the epidermal mucosa as a phage enrichment layer. To this end, we analyzed the microbial content of the skin mucus of wild and farmed eels by using a metagenomic approach. We found a great abundance of replicating phage genomes (concatemers) in all the samples. They were assembled in four complete genomes of three Myovirus and one Podovirus. We also found evidences that ΦKZ and Podovirus phages could be part of the resident microbiota associated to the eel mucosal surface and persist on them over the time. Moreover, the viral abundance estimated by epiflorescent counts and by metagenomic recruitment from eel mucosa was higher than that of the surrounding water. Taken together, our results support the hypothesis that claims a possible role of phages in the animal mucus as agents controlling bacterial populations, including pathogenic species, providing a kind of innate immunity.

## INTRODUCTION

Animals, including humans, protect their mucous membranes by covering them with a mucus layer that contains microcidal and microstatic compounds produced by epidermis-associated immune cells. Being permanently immersed, fish mucus attracts aquatic bacteria, including pathogens, some of which have chemotactic systems that “sense” mucin, glucids, and other nutrients present in mucus (Valiente et al., 2008). For this reason, fish exposed surfaces are covered by a dense mucus layer enriched in antimicrobial compounds, most of them uncharacterized (Ellis, 2001). While most aquatic bacteria are supposed to be controlled by mucus antimicrobial compounds, pathogens have developed mechanisms to resist their attack and colonize mucosal surfaces.

Recently, Barr et al. (2013a,b) proposed a hypothesis suggesting an additional protective function for the mucus: to trap bacteriophages in order to detect and destroy invading bacteria before they reach to the epithelium. The attraction of virus to mucus surfaces has been corroborated in coral mucus using enumeration techniques (Nguyen-Kim et al., 2014). If this hypothesis is true, phages could play a significant role in defense against aquatic pathogens. However, the study of phages present in environmental samples is not easy. Traditional approaches are very limited as they rely on previous knowledge about the host and its culture. On the other hand, metaviromic approaches are constrained by the low amount of viral DNA in natural samples that forces the use of heavily biased amplification steps (Minot et al., 2011; Phan et al., 2011; Hurwitz and Sullivan, 2013; Zablocki et al., 2014). However, metagenomic DNA (derived from the cellular fraction) is known to contain a high proportion of phage DNA. Caudovirales lytic phages replicate forming genome concatamers that can be larger than the cell genome and provide an ideal subject for direct sequencing and assembly (Mizuno et al., 2013; Rodriguez-Valera et al., 2014).

The objective of the present study was to test the hypothesis of Barr et al. (2013a,b) by using the European eel (*Anguilla anguilla*) as a model fish and the metagenomic approach mentioned above to identify the viruses present in the mucus. Eels are quite resistant to stressors and are well equipped against pathogens (Ellis, 2001). Their lack of protective macroscopic scales is compensated with a thick mucus layer especially rich in antimicrobial compounds (Tesch, 2003). We postulate that this protection is partially due to the mucus-attracted phages. We have sampled the mucus from wild eels captured in three Mediterranean wet lands (Albufera Lake, Ebro Delta, and Cabanes; Figure S1). In addition, the European eel is also a commercially important species in Europe, Asia, New Zealand, and USA, that is produced in farms, although its life cycle has not been closed and eel farmers fatten juvenile glass eels caught from natural stocks (Lee et al., 2003). Therefore, we have used also farmed eels, and used a metagenomic approach to identify replicating phages. We have analyzed the water metagenome from one of the sampling sites (Ebro Delta) for comparison. We describe the genomes of four complete, abundant phages (three Myoviruses and one Podovirus), plus one incomplete myovirus, that were directly assembled from the metagenomic reads. This is the first report of abundant phages in the epidermal mucosa of eels or any other fish species.

## MATERIALS AND METHODS

### SAMPLING

Three natural parks in the Mediterranean Spanish coast were chosen to fish wild eels: Albufera de Valencia (Valencia, 39°19′54′′N, 0°21′8′′W), Alfacada pond (40° 40′ 45.609′′N, 0° 49′ 52.7982′′W) in the Ebro delta, two samples a year apart (2013–2014), and the Prado Cabanes (Castellón 40°12′03.5′′N 0°12′26.4′′E; Figure S1). Moreover, a volume of 25 L of water from Ebro delta (2014) was also analyzed.

To recover eel mucus the animals were kept in a fish bowl with 1 L of PBS (1% NaCl) during 15–20 min. Previous experience has shown that 50–100 ml of mucus is released by this procedure by 5–10 adult animals. The PBS was then filtered sequentially through 5, 1, and 0.22 μm using a peristaltic pump. After that, 0.22 filter was treated using 1 mg/ml lysozyme and 0.2 mg/ml proteinase K (final concentrations) and total DNA was extracted with phenol/chloroform/isoamyl alcohol and chloroform/isoamyl alcohol, and DNA integrity was checked by agarose gel electrophoresis (Ghai et al., 2012).

### ASSEMBLY AND GENERAL CHARACTERIZATION OF PHAGE GENOMES

We have sequenced by Illumina Hiseq 2000 using pair-end technology the DNA from the microbiota present in the epidermal mucosa of eels captured in Ebro Delta and Cabanes, and also the water sample, and by 454 FLX sequencer (Roche, Basel, Switzerland) the microbiota of mucus from eels, captured in Albufera de Valencia, farmed, and glass eels. These metagenomes have been deposited in NCBI SRA under the following accessions: SRR1578065, SRR1578068, SRR1578098, SRR1580820, SRR1580821, and SRR1580823. Metagenomic reads were assembled using Velvet (k–mer 51) and were annotated using Prodigal (Zerbino and Birney, 2008; Hyatt et al., 2010; Table S1). Only contigs bigger than 1 kb were considered for analysis. Annotation was refined manually using HHpred (Söding, 2005). The largest phage contigs were identified manually after annotation of contigs. The rest of them were fished using these contigs on a BLASTN search (Altschul et al., 1997). Moreover, CRISPR approach (see below) allowed us to find the prophage ProEnteroC171.

Phage genomes, classified as different families using ICTV, were downloaded from NCBI to accomplish a genomic comparison with our genomes. We used a previous protocol described (Mizuno et al., 2013). Comparisons were done using tBLASTX and BLOSUM45 matrix (Altschul et al., 1997). Minimum 30% sequence identity, 30 aa length and a maximum *e*-value of 10^-3^ were considered to filter the results. Phylogenetic tree was constructed using PHYLIP package (Felsenstein, 1993).

To find out if these contigs were closed, direct terminal repeats for phages were determined using Uniprot UGENE program (Okonechnikov et al., 2012). BLASTP search was used to identify the genes of interest to build the trees and the Ig-like domains. *Pseudomonas* phage phiKZ was used as the query in the ΦKZ genus specific searches. Finally, phylogenetic analyses using a group of genes (terminase large subunit, RNA and DNA polymerase [RNApol and DNApol], ribonucleoside diphosphate reductase alpha chain [*nrdA*], and phosphate starvation-induced protein [*phoH*]) allowed the taxonomic classification of these contigs. Trees were done using FastTree with 100 bootstraps (Price et al., 2009). All genes and genomes were download from Genbank, Pfam, or Uniprot database (Benson et al., 2005; Finn et al., 2006; Consortium, 2014).

### HOST IDENTIFICATION

In order to identify the potential host for these phages CRISPR approach and tetranucleotide usage pattern (TUP) have been proved (Stern et al., 2012; Ogilvie et al., 2013). On one hand, three different approaches were used to search for putative CRISPR cassettes. (1) Protocol described by Stern et al. (2012) was tested, it consists in inferring the spacers using known CRISPR repeats and use these spacers to fish phage assembled contigs. To filter false positive spacers a minimum of 75 bp read length and 80% query coverage were used and to consider a spacer in a contig 100% identity and coverage were used. (2) Spacers were searched directly from assembled contigs using spacers from CRISPR database. Only matches over 80% coverage and 90% identity were considered. (3) CRISPRfinder tool was used to find CRISPR cassettes in assembled contigs.

One the other hand, spacers detected from different approaches were used to find viral contigs and putative hosts for those contigs. BLASTN search was done considering 100% identity as a filter. Finally, TETRA 1.0 was used to identify contigs with similar TUP (Teeling et al., 2004). Only contigs bigger than 10 kb and values of 0.6 or over were considered for analysis.

### ABUNDANCE

In order to compare the abundance of viral and bacterial population in our datasets we count the number of reads recruited to viral and bacterial concatenated contigs. The number of reads was calculated using BLASTN and considering a minimum identity of 95 and a maximum *e*-value of 10^-3^ for filtering the results. The number of reads recruited against the genomes assembled was normalized per the size of the genome or concatenated (kb) and the dataset (Gb).

The abundance of those phages in other niches was analyzed comparing by TBLASTN the reads of marine (GOS, Albufera, Sargasso sea, Tampa bay, and Mediterranean bathypelagic habitat) and animal associated (mouse, termite, canine, cow, coral, and human) metagenomes against the viral proteins isolate in our data. Metagenomes were download from MG-RAST server (Meyer et al., 2008). A minimum identity of 60% and 10^-5^ *e*-value was considered for filtering the results. MG-RAST ID: 4440414.3, 4440440.3, 4440439.3, 4440413.3, 4440424.3, 4440422.3, 4440412.3, 4440411.3, 4440066.3, 4440062.3, 4440055.3, 4440056.3, 4440065.3, 4440063.3, 4440059.3, 4440064.3, 4483775.3, 4450680.3, 4450678.3, 4440284.3, 4440283.3, 4440285.3, 4440286.3, 4440102.3, 4444702.3, 4444703.3, 4444165.3, 4444164.3, 4440373.3, 4440375.3, 4440379.3, 4440377.3, 4440374.3, 4440381.3, 4440376.3, 4440378.3, 4440371.3, 4440370.3, 4440380.3, 4440372.3, 4447454.3, 4447455.3, 4447456.3, 4447457.3, 4447446.3, 4447447.3, 4447448.3, 4447449.3, 4441025.3, 4442464.3, 4441625.3, 4441625.4 4441627.3, 4441623.3, 4441624.3, 4441621.3, 4441622.3, 4441629.3, 4441628.3, 4441626.3, 4440330.3, 4440951.3, 4472804.3, 4472821.3, 4473347.3, 4473348.3, 4473365.3, 4473372.3, 4473378.3, 4473389.3, 4473411.3, 4473417.3, 4473438.3, and 4478542.3.

We also used VIROME database to search in the uploaded viromes for annotated ORFs to any of the viral genus found in our metagenomes (ΦKZ, ΦKMV, FelixO1like, and Φ16). The number of ORFs was count using the online available tools in the VIROME website (Wommack et al., 2012).

### PHAGE COUNTS

Samples from surrounding water and epidermal mucosa of eels farmed in tanks at 22°C and 1% salinity in facilities at University of Valencia (Planta de Acuarios de Experimentación, PAE) were collected. Samples were maintained on ice, sonicated in 3 pulses during 4 s. 50 and 3 ml of water and mucus, respectively, were directly filtered per 0.02 μm Anodisc polycarbonate filter (Whatman). Anodisc filters were stained with SYBR Green 5x, washed and visualized using epifluorescence microscope. For each sample, 25–30 images were analyzed using ImageJ (Schneider et al., 2012). Counts of bacteria and virus-like particles per milliliter were made using a previous protocol described (Patel et al., 2007).

## RESULTS

We have previously analyzed the bacterial population present in the epidermal mucosa of farmed, wild, and glass eels by analysis of their metagenomes, which provided a first glimpse into the microbiota of the epidermal mucosa of eels (Carda-Diéguez et al., 2014). Interestingly, clear differences were observed in bacterial composition depending on the origin and the conditions where eels lived. Thus, *Pseudomonas* appeared as a common genus in eels and glass eels independently of their origin. In addition, *Vibrio*, *Shewanella*, *Aeromonas*, *Stenotrophomonas*, and *Acinetobacter* were the most abundant genera present in the microbiota from wild eels, while *Comamonas*, was only found in farmed eels (Carda-Diéguez et al., 2014).

Summary statistics for the sequenced metagenomes are provided in Table S1. The assembly of the Illumina datasets yielded a total of 17 viral contigs >10 kb and 23 contigs <10 kb, which were clearly related (>98% nucleotide identity) to the longer contigs (Figure S2). We selected five of these contigs for further analysis. From these metagenomes, we also assembled several contigs that appeared nearly identical to known bacterial genomes. Figure S3 shows an 893 kb contig that is >95% identical along its entire length to the genome of *Pseudomonas aeruginosa PA14* [originally isolated from a human patient (Lee et al., 2006)]. Long fragments of Caudovirales genomes have been detected in cellular metagenomes, probably due to the concatamer formation during lytic cycle, a natural process of genome amplification (Mizuno et al., 2013). In order to check if any of the assembled phages represented complete genomes, we performed all *vs* all comparisons of these viral contigs. We detected contigs overlapping in a circular fashion in the metagenome datasets from the 0.22 and 1 μm filters of the Ebro Delta (See Figure S2). This result suggests that all the genes in the genome had been captured and that these contigs represent complete phage genomes. Another method that may be useful in detecting the completeness of genomes is the presence of terminal repeats within the same contig. We identified a total of 4 complete phage genomes by these methods (See **Table 1** for details). Their analysis revealed that all correspond to tailed-bacteriophages. Additionally, nine putative prophages were also identified by the presence of host genes at one or both ends of the contig being their hosts *Stenotrophomonas*, *Achromobacter*, and *Enterobacteriaceae bacterium 9_2_54FAA* (Table S2 and Figure S4). Since we had multiple metagenomes, we checked for redundant contigs and found several examples (Figure S5). Even samples taken one year apart yielded identical phage contigs indicating a remarkable resilience and conservation.

**Table 1:** Complete sequenced phages properties.

	Contig	Length (bp)	Genus	%GC	#ORF	%Annotated ORF
Cabanes	MyoΦKZC1	220.117	ΦKZ	45,19	227	32,15
Ebro Delta	MyoΦKZC12	313.980	ΦKZ	58,22	281	79,60
	MyoC35	116.788	FelixO1like	34,72	92	43,81
	PodoΦKMVC113	42.235	phiKMV-like (Podoviridae)	59,28	28	54,90
	MyoC197	40.198*	Unclassified Myoviridae	60,95	58	57

*incomplete genome.

### MYOVIRUSES

**FIGURE 1:**
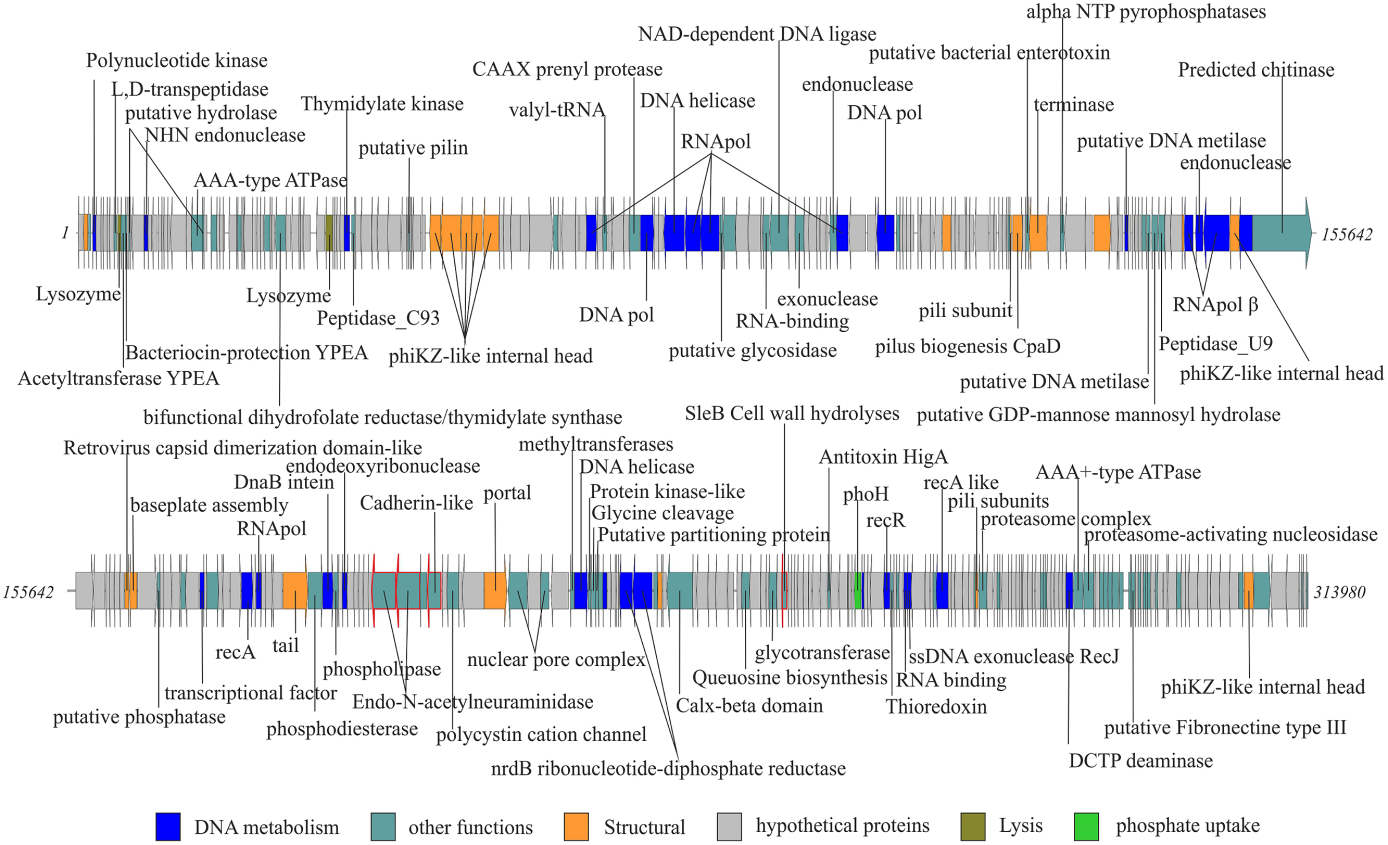
**MyoΦKZC12, complete new phage genome.** Complete genome of the longest phage is represented in two rows. Gene functions ORFs are colored according to the color code. Ig-like domains are highlighted using a red fringe.

**FIGURE 2:**
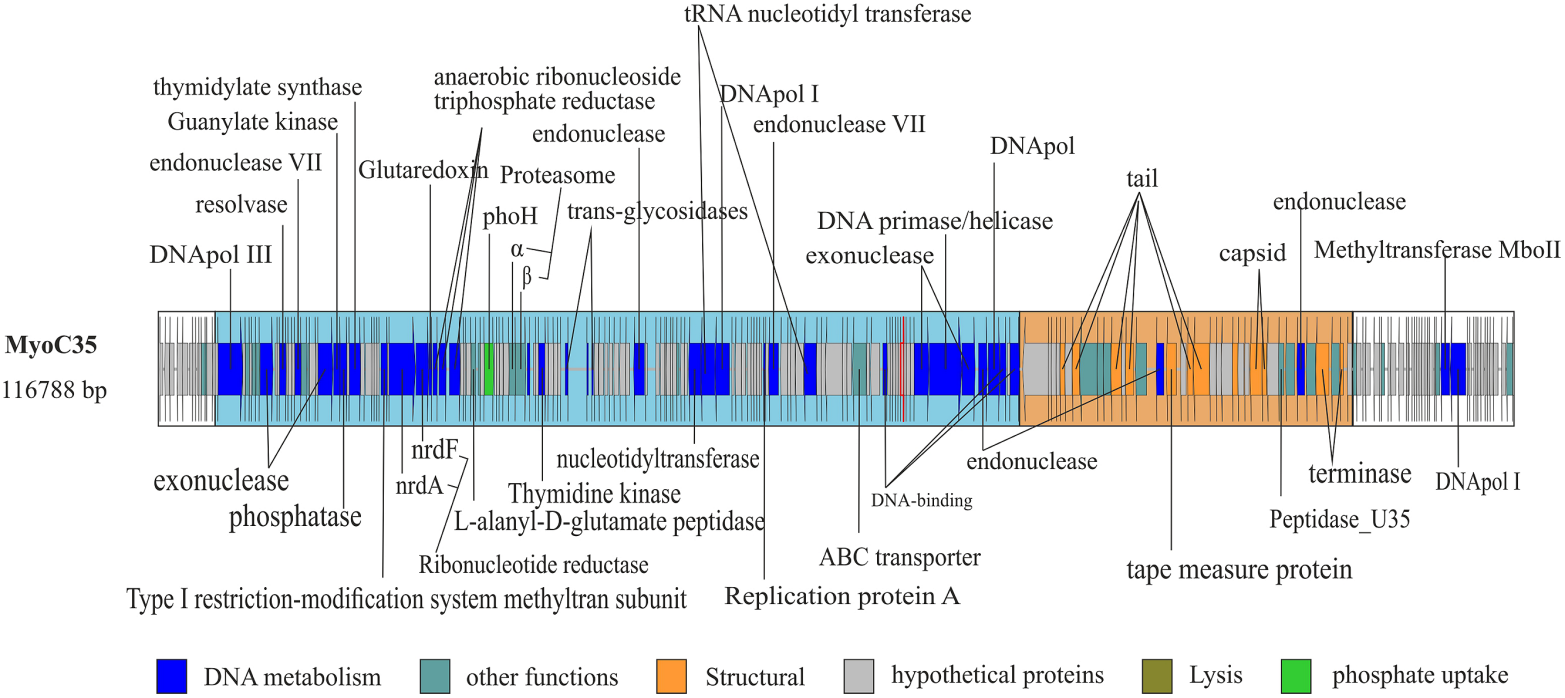
**Complete genome of MyoC35.** Functional domains were highlighted using boxes colored according the function of the genes. White box indicate no functional domain. Ig-like domains are highlighted using a red fringe.

**FIGURE 3:**
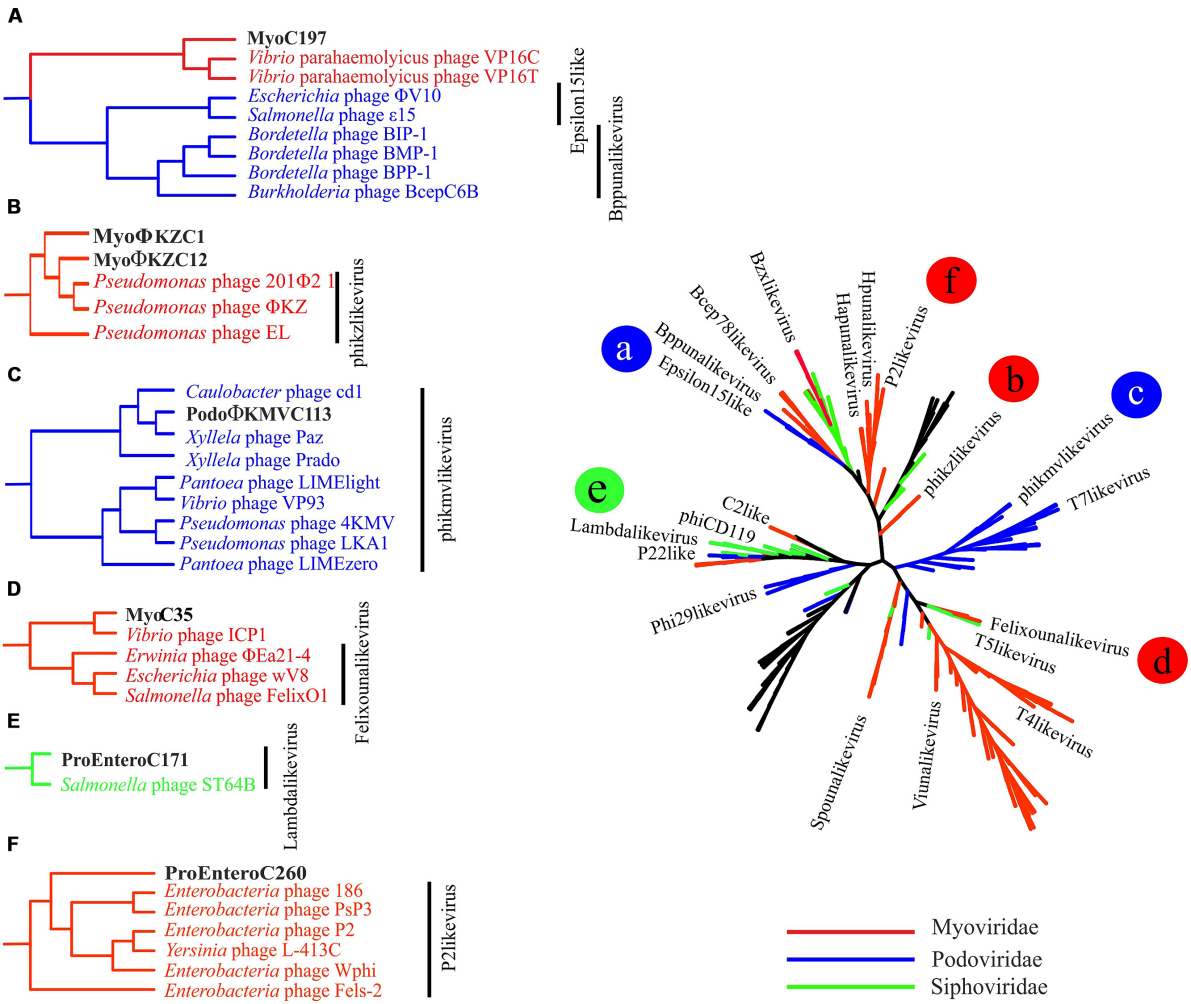
**Complete genomes phylogeny.** All-vs-all comparison using NCBI phages genomes and the ones sequenced in our metagenome. The clustering was based on a sequence similarity derived metric. Colors were used to differentiate viral families. Branches in black indicate unclassified genomes. The branches in which the new complete genomes clustered are shown in detail on the left and marked with letters **(A–F)** that correspond with the position in the tree.

Among tailed bacteriophages, Myoviruses are known because of their large sizes ranging from 11.6 to 358.6 kb. We found three Myoviruses that we named MyoΦKZC1, MyoΦKZC12, and MyoC35, with genome sizes of 220, 313, and 116 kb, respectively, (**Table 1**; **Figures 1 and 2**; Figure S6). MyoΦKZC12 and MyoΦKZC1 shared a protein with a putative CAAX protease domain and MyoC35 presented a putative ubiquitin-ligase, and the alpha and beta subunits of a proteasome complex. By complete genome analysis, MyoΦKZC12, and MyoΦKZC1 were predicted to be part of the ΦKZ genus (**Figure 3**). This genus is notorious for their genes involved in nucleotide metabolism (e.g., thymidylate synthase, thymidylate kinase, ribonucleoside diphosphate reductase subunit beta [*NrdB*] and alpha [*NrdA*], and dihydrofolate reductase, *RuvC* Holliday junction resolvasome; Cornelissen et al., 2012; Jang et al., 2013) all of which were found in MyoΦKZC12 and MyoΦKZC1 genomes. Phylogenetic analysis using DNApol (Figure S7), terminase large subunit (Figure S8) and ribonucleoside diphosphate reductase alpha chain (*nrdA*; Figure S9) genes also supported the taxonomic classification. The GC content of MyoΦKZC1 was in the expected range and MyoΦKZC12 reached the highest value ever published for this genus (58.22%; Table S3). Genomic comparison between *Pseudomonas* phage ΦKZ, PA7, and MyoΦKZC12 and MyoΦKZC1 revealed a low structural conservation together with a low nucleotide identity (Figure S6). In fact, only some structural protein, DNApol, RNApol, and terminase showed homology (30–60% aminoacid identity). Previous studies have shown a high rate of divergence among members of this genus (Cornelissen et al., 2012; Jang et al., 2013). Representatives of this genus target a variety of Gram-negative bacteria such as *Pseudomonas*, *Vibrio*, *Yersinia*, *Cronobacter*, *Salmonella*, and *Erwinia* (Table S3). We could not assign a putative host for these Myoviruses. Recent studies have shown that the eight RNA polymerase subunits from *Pseudomonas* phage ΦKZ form two polymerases: virion (vRNAP) and non-virion RNA polymerase (nvRNAP; Ceyssens et al., 2014). Moreover, Ceyssens et al. (2014) suggested that all subunits are present in all phages of the genus and the transcription is completely independent from the host. We have been able to identify some of them in MyoΦKZC12 and MyoΦKZC1 genomes. Further phylogenetic analysis using all RNApol subunits found in each ΦKZ genome showed a clustering of the eight different subunits in the tree (Figure S10).

MyoC35 (**Figure 2**, 116 kb) was classified as a FelixO1like phage by both whole genome comparison against NCBI (**Figure 3**) and clustering according to the terminase gene phylogenetic tree (Figure S8). In both analyses, MyoC35 clustered with *Cronobacter* phage vB_CsaM_GAP31 and *Vibrio* phage ICP1 although showed a low structural conservation and only DNApol, NrdA, and some structural proteins were similar (<50% identity; Figure S11). Those phages have not been assigned to any genus within the Myoviridae family. *Vibrio* phage ICP1 has been linked to FelixO1like genus by homology analysis using NrdA (Seed et al., 2011). Phylogenetic analysis using this gene clustered MyoC35 close to *Vibrio* phage ICP1 (Figure S9). However, the same analysis using DNApol clustered MyoC35 close to the Φ16 branch, a group recently added to NCBI which also appeared in this niche (see below; Figure S7). *Vibrio* phage ICP1 is a very specific *Vibrio* phage which has been isolated from stool samples from a cholera patient (Seed et al., 2011). In Ebro Delta metagenome, only small contigs were annotated as *V. cholerae*, however, *Vibrio* sp. *RC341* [closely related to *Vibrio cholerae* and *V. mimicus* (Haley et al., 2010)] was one of the most abundant organisms by number of contigs. They could be representatives of MyoC35 host.

Interestingly MyoΦKZC12 and MyoC35 genomes harbor a *phoH* gene, an auxiliary metabolic gene implicated in the regulation of phosphate uptake and metabolism under low-phosphate conditions (Hsieh and Wanner, 2010). The inclusion of this gene in the phylogenetic analysis supports the value of *phoH* as a signature gene for marine viruses (Figure S12; Goldsmith et al., 2011).

**FIGURE 4:**
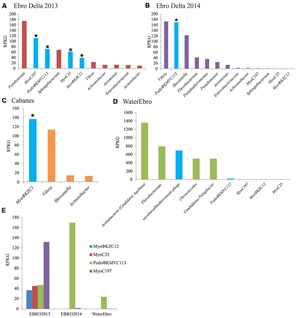
**Bacterial and viral abundance in the metagenomes.** The abundance of bacterial genera and viral genomes were calculated by normalizing the number of reads recruited in their respective metagenomes per the size of the concatenated contigs (Kb) and the gigabases of the dataset (RPKG). Bacteriophage counts were highlighted in blue. Complete genomes were marked with an asterisk. **(A)** Ebro Delta 2013; **(B)** Ebro Delta 2014; **(C)** Cabanes-Torreblanca; **(D)** Water from Ebro Delta 2014; **(E)** Abundance of each phage genome in their respective metagenome; EBRO2013 and EBRO2014, metagenomes from Ebro Delta in 2013 and 2014, respectively; Water Ebro; metagenome from the water surrounding the eels from 2014.

MyoC197 was predicted to be an incomplete myovirus with mixed characteristics (Figure S13, 40.2 kb). On one hand, this phage clustered with two *Vibrio* phages (VP16C and VP16T) by several phylogenetic analyses (**Figure 3**; Figures S7 and S8) although lacking the polypeptide deformylase gene and *vapE* (virulence associated protein), two typical genes of these *Vibrio* phages (Seguritan et al., 2003). On the other hand, MyoC197 contained several lambda phage related genes (genes for lambda head decoration protein D, major capsid protein E, and terminase large subunit GpA) and some Mu-like viruses related genes (genes for Mu-like prophage tail sheath protein, phage tail tube protein, FluMu protein Gp41, Mu-like prophage DNA circulation protein, and an uncharacterized protein conserved in bacteria DUF2313). In fact, 25 of 58 genes in the contig had the highest similarity to *Vibrio* phage genes (50–70% identity; Figure S14). The *Vibrio* phages came from an environmental isolate of *V. parahaemolyticus* and are now classified as a new genus named Φ16 (Seguritan et al., 2003). Interestingly, MyoC197 was one of the most abundant viruses recruited from the Ebro Delta metagenome in 2013 (**Figure 4E**). Contigs annotated as *Vibrio* in metagenome from eels fished in Ebro Delta were assigned to *Vibrio sp. RC341*, *V. anguillarum*, *V. vulnificus*, and *V. fischeri* suggesting those species as putative hosts for MyoC197.

### PODOVIRUSES

According to the phylogenetic analysis from the complete genome, PodoΦKMVC113 (Figure S13, 42.2 kb) is a Podovirus from the Autographivirinae subfamily and a putative new member of the ΦKMVlike genus of T7 related phages infecting *P. aeruginosa* (40.7–44.5 kb; **Figure 3**). RNApol, a hallmark gene for this genus, was found in PodoΦKMVC113, while single-strand interruptions (nicks) also typical in this genus were not present (Kulakov et al., 2009). Annotation results showed several genes assigned to other phages such as *Caulobacter* phage Cd1 and *Xanthomonas* phages. Phylogenetic analysis using the terminase and RNApol genes clustered PodoΦKMVC113 with *Caulobacter* phage Cd1, *Xylella* phages Paz, and Prado, all of them members of ΦKMVlike genus (Figures S8 and S10). As the other ΦKMV phages, PodoΦKMVC113 follows the structural organization based on three functional gene clusters encoded on the forward strand but with a rearrangement of lysis and structural domains (data not shown). A proteasome alpha subunit was also identified in this genome.

The segregation of the branch of PodoΦKMVC113, *Caulobacter* phage Cd1, and *Xylella* phages Prado and Paz suggests the formation of a new group very close to the ΦKMV genus (**Figure 3**). Interestingly, PodoΦKMVC113 was completely assembled in the same sampling point (Ebro Delta) a year later (Figure S4; identity 100%). Although no contig similar to PodoΦKMVC113 was assembled in the metagenome from the water, several reads were recruited against this genome (**Figure 4E**). It is probable that the complete genome would be assembled with larger sequencing efforts.

### PHAGE ABUNDANCE IN MUCUS AND WATER AND PRESENCE OF SIMILAR PHAGES IN OTHER HABITATS

In order to assess the relative abundance of these phages in the different metagenomes, the contigs were compared with the raw reads. In all cases, the viral contigs recruited more than bacterial contigs except for *Pseudomonas* and *Sphingobium* in the sample from Ebro Delta (**Figures 4A,B**). In Cabanes, where the bacterial population was calculated to be composed by 60–80% of *Vibrio*, the number of reads recruited to MyoΦKZC1 genome was higher than those recruited to bacterial representatives (**Figure 4C**). Moreover, when these proportions were compared with the water metagenome, the relative abundance of phages and bacteria were reversed, suggesting that bacteriophages were retained or continuously produced in the epidermal mucosa (**Figure 4D**).

To test this apparently higher abundance of phages in mucus samples, we directly counted phages from mucus of farmed eels and the surrounding water by epifluorescence. As expected, the abundance of phages in mucus samples (3.14⋅10^6^ virus-like particles/ml) was higher than in the surrounding water (1.47⋅10^5^ virus-like particles/ml) confirming that mucus concentrates phages present in the water. We also counted bacteria and compared the ratio virus/bacteria in the mucus and water samples. Bacteria were more abundant in mucus (1.62⋅10^6^ vs. 1.49⋅10^4^ bacteria-like particles per ml of mucus or water, respectively), suggesting that bacterial population was even more concentrated in the eel secretion. This led to a lower phage to bacteria ratio in the mucus (ca. 2:1) than in the water (ca. 10:1). Barr et al. (2013a) found 4.4 higher ratios of phages to bacteria in the mucus than in the surrounding environment (on average) in a study that included one teleost surface mucus. It is possible that our method to collect mucus by passive release from the fish (rather than by suction device as done in the mentioned paper) might have affected the results.

The relative abundance of phages in their respective samples was analyzed by recruitment as well. On one hand, MyoC197 was the genome that recruited the most while PodoΦKMVC113, -C35, and -C12 reached similar but lower levels from Ebro Delta in 2013 (**Figure 4E**). On the other hand, a year later, PodoΦKMVC113 was the only one that increased its recruitment level while the Myoviruses almost disappeared. Considering that the bacterial population changed in this last sample, probably as a reflection of a change in water salinity (5.52–17.7 mS/cm; **Figures 4A,B**), the persistence of PodoΦKMVC113 and MyoC197 in the epidermal mucosa of eels in Ebro Delta for a year suggests that the host of these phages had remained in the epidermal mucosa while the host of the others Myoviruses did not. The bacterial genus that maintained and even raised its proportion in the epidermal mucosa through time was *Vibrio*. Therefore, this result suggests that *Vibrio* could be the host of PodoΦKMVC113 and MyoC197. Accordingly, the great reduction of the others Myoviruses mentioned could be related to a reduction in the host population. The decreasing numbers of reads recruited by *Pseudomonas, Achromobacter*, and *Sphingobium* make us consider them as putative hosts for these phages (**Figures 4A,B**). Finally, the only phage which recruited a significant number of reads in the metagenome from the water sample was PodoΦKMVC113.

Finally, metagenomes and metaviromes from marine and animal associated habitats were downloaded from MG-RAST in order to look for these phages in other niches. None of these datasets gave a number of matches high enough to consider that a similar virus was present in the searched metagenomes except for canine feces and cow rumen metagenomes in which reads similar to the ΦKZ and ΦKMV representatives were found. When reads identified as DNApol or RNApol were included to probe the presence of these genera in the rest of metagenomes, a single DNApol found in the cow rumen metagenome was similar to MyoΦKZC12 and MyoΦKZC1 and it clustered in the expected branch (Figure S7). Finally, two RNApol genes were recovered similar to MyoΦKZC12 from a canine metagenome and one to PodoΦKMVC113 from a cow metagenome that clustered in the respective branch (Figure S10). Moreover, we searched in the VIROME database for annotated open reading frames (ORFs) of the different viral genus found in epidermal mucosa. Members of ΦKMV, FelixO1likevirus, and Φ16 were found in practically all marine, soil, and host-associated viromes while ΦKZ genus was present in very low amounts in some marine viromes but was highly represented in host-associated ones, especially in the cow rumen virome. ΦKZ members have been isolated from bacterial pure cultures from a great diversity of environments: sewage, pond water, compost, soil, chicken feces, fresh, and marine water, but this is the first time that they have been sequenced from an environmental habitat. It is noteworthy that 40 contigs from Albufera metagenome were annotated as ΦKZ (30–60% identity). Furthermore, DNApol and RNApol subunits were found in some of these contigs and included in the analysis confirming the annotation (Figures S7 and S10). This turned ΦKZ in the only genus found in all three metagenomes from mucus of wild eels.

## CONCLUSION, VIRAL POPULATION, AND FUNCTIONAL ROLE

Most of the metagenomic studies have been carried out with human or water related environments (Venter et al., 2004; Daniel, 2005; Ghai et al., 2011, 2012; Yu and Zhang, 2012; Lipson et al., 2013; Mizuno et al., 2013). There are only few studies of the microbiota associated to animals and even less to wild animals (Vega Thurber et al., 2009; Swanson et al., 2011; Marcobal and Sonnenburg, 2012; Parfrey and Knight, 2012; Ross et al., 2012, 2013; Schwartz et al., 2012; Singh et al., 2012; Wong and Rawls, 2012; Bodewes et al., 2014). In this study, we analyzed the viral content of the epidermal mucosa of the eel to test the hypothesis that it concentrates phages present in water, which in turn, control bacterial populations, including those of pathogenic species. We found a replicating viral community in this niche formed by Myovirus and Podovirus. In addition, we have found evidences that ΦKZ genus and the Podovirus could be part of the resident microbiota associated to the eel mucosal surface.

The recovery of large contigs of genomes from metagenomic samples is normally an indication of lower diversity. In this sense, the assembly of large genomic tracts from this animal mucus samples indicates low diversity what in itself is remarkable since this external layer is immersed in waters with rich microbial communities. If it actually is a defense mechanism for the protection of the fish it remains to be demonstrated. On the other hand, we found evidence for large amounts of phages. There is little doubt that the genomes retrieved derive from actively replicating phages in cells present in the samples. In addition, despite the abundance of potential pathogens found in the metagenomes (especially, in wild eels: *Pseudomonas aeruginosa*, *Aeromonas veronii, V. anguillarum, Acinetobacter baumanii, Achromobacter xylosoxydans*, etc) we did not observe clinical symptoms of infectious disease in the captured eels.

All these findings support the BAM model described by Barr et al. (2013a) that proposes that phages attached to the mucous are ideally located to infect bacterial cells attracted by the rich nutrients provided by the animal. Although epifluorescent counts gave in our case lower phage/bacteria ratios in the mucus that in the water, we could detect a clear enrichment of phages in the mucus (20 times more). The passive way to obtain mucus that does not probably retrieves the mucus layers most closely associated to the fish surface might explain this discrepancy.

Phages use Ig-like domains present in the capsids to attach to mucin. However, only a single putative Ig-domain was found in MyoΦKZC12 and a fibronectin type III domain in MyoΦKZC1 phage. Considering that the Barr study was performed in T4 group bacteriophages and MyoΦKZC12 and MyoΦKZC1 are members of a genus classified as T4-like, it is possible that attachment through Ig-like domains is specific of T4 bacteriophages and the rest use a different protein. When others Ig-like domains and domains that are known to bind to carbohydrates, which could mediate BAM-mechanisms were searched in the sequenced phages, a C-type lectin domain was found in MyoΦKZC1 and MyoC35. Moreover, three and one Ig-like domains annotated in Pfam as invasion/intimin cell adhesion were found in MyoΦKZC12 and MyoC197, respectively. Finally, we found BACON domain (Bacteroidetes-Associated Carbohydrate-binding Often N-terminal) in a tail protein of MyoC197.

Recent work related to bacterial infection in aquaculture proposes phage therapy as a preventive strategy (Vinod et al., 2006; Mateus et al., 2014; Rong et al., 2014). Along these lines, phage therapy using mucus associated phage communities against potential pathogens could be a useful concept in European eel farming.

## Conflict of Interest Statement

The authors declare that the research was conducted in the absence of any commercial or financial relationships that could be construed as a potential conflict of interest.
