# Supplementary material for: Replicating phages in the epidermal mucosa of the eel (Anguilla anguilla)
Source: Front Microbiol. 2015 Jan 29;6:3. doi: 10.3389/fmicb.2015.00003 (PMC4310352; doi:10.3389/fmicb.2015.00003)
Supplement: Supplementary file 1 [file Data_Sheet_1.PDF]

Supplementary Material

Supplementary Figures and Tables

1. Supplementary Tables

Supplementary Table 1. Dataset summary. The sequencing and assembling results are summarized in the table.

Sequencing platform	454			Illumina				
Sampling points	Farmed eels	Glass eels	Wild eels Albufera	Wild Ebro Delta (2013)	Wild Ebro Delta	Wild Cabanes	Wild Ebro Delta (2014)	Ebro Delta Water
Filter size (µm)	0.22	0.22	0.22	0.22	1	0.22	0.22	0.22
Dataset size (Mb)	49.8	70	45.3	3666	3939	1300	3622	959
Number of sequences	91574	101780	70510	23541344	25287346	12325876	46064452	12490702
Average length (bp)	486,1	634,6	587,5	87.6	88.25	87.4	78.6	76.9
Number of contigs >1Kb (Mb)	8217 (16.6)	6074 (8.2)	1609(20.8)	6554 (39.58)	5050 (45.13)	1016 (23.08)	12069 (35.8)	2608 (6)
Average contig length (bp)	2027.7	1354	1291.5	6040.1	8938	2272	2970.2	2302
Number of contigs >10Kb (Mb)	0	0	0	622 (24.28)	699 (33.26)	7 (0.3)	415 (11.2)	27 (0.56)
Average contig length (bp)	0	0	0	39041.8	47590.1	43310.6	27052	20993
Viral contigs (Mb)	67 (0.12)	218 (0.30)	79 (0.11)	6 (0.51)	33 (0.43)	1 (0.22)	181(0.86)	127(0.6)
Average contig length (bp)	1890	1399.6	1413.8	85931	13066.2	220068	4786.9	4770.9
Viral contigs > 10Kb (Mb)	0	0	0	4 (0.50)	12 (0.36)	1	21 (0.49)	14 (0.39)
Average contig length (bp)	0	0	0	126083.5	29921.8	220068	23795.6	27867.9

Supplementary Table 2. Genomic properties of prophages sequenced.

Contig	prophage length (kb)	GC%	Host
ProStenoC191	41068	68.3	*Stenotrophomonas*
ProAchroC45	42197	63.4	*Achromobacter*
ProAchroC186	33297	63.4	
ProEnteroC6	30621	48.1	*Enterobacteriaceae bacterium*
ProEnteroC38	20823	46.79	
ProEnteroC171	34456	47.3	
ProEnteroC260	23048	49.6	
ProEnteroC286	16618	44.2	
ProEnteroC299	25725	53.1	

Supplementary Table 3. Genomic properties of ΦKZ members.

PhiKZlike	Length (bp)	%GC	ORF	tRNA	RNApol subunits
Pseudomonas phage EL	211215	49.3	201	1	6
Pseudomonas phage phiKZ	280334	36.8	369	6	8
Pseudomonas phage 201phi2-1	316674	45.3	461	1	7
Pseudomonas phage PhiPA3	309208	47.43	375	3	5
Pseudomonas phage OBP	283757	43.5	309	4	8
Halocynthia phage JM-2012	167292	35.4	163	0	8
Erwinia phage phiEaH2	243050	51.28	262	11	8
Yersinia phage phiR1-37	262391	32.4	367	4	7
Cronobacter phage CR5	223989	50.1	231	0	7
Pseudomonas phage PA7	266743	36.96	337	7	8
Salmonella phage SPN3US	240413	48.54	264	2	6

2. Supplementary Figures

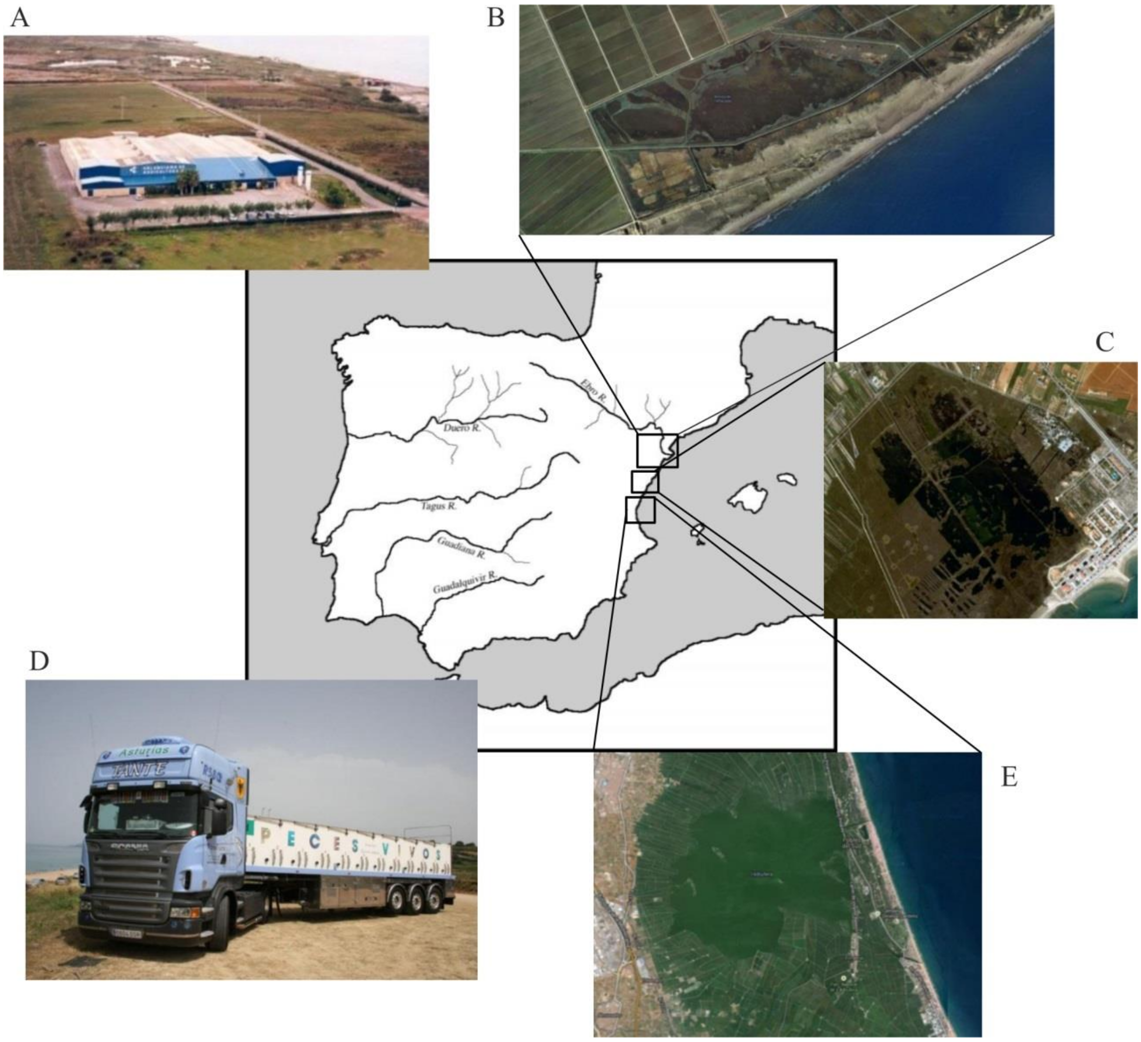

Supplementary Figure 1. Sampling points in the Mediterranean coast. A, fish farm; B, Ebro Delta; C, Cabanes; D, track were glass eels are transported; E, Albufera.

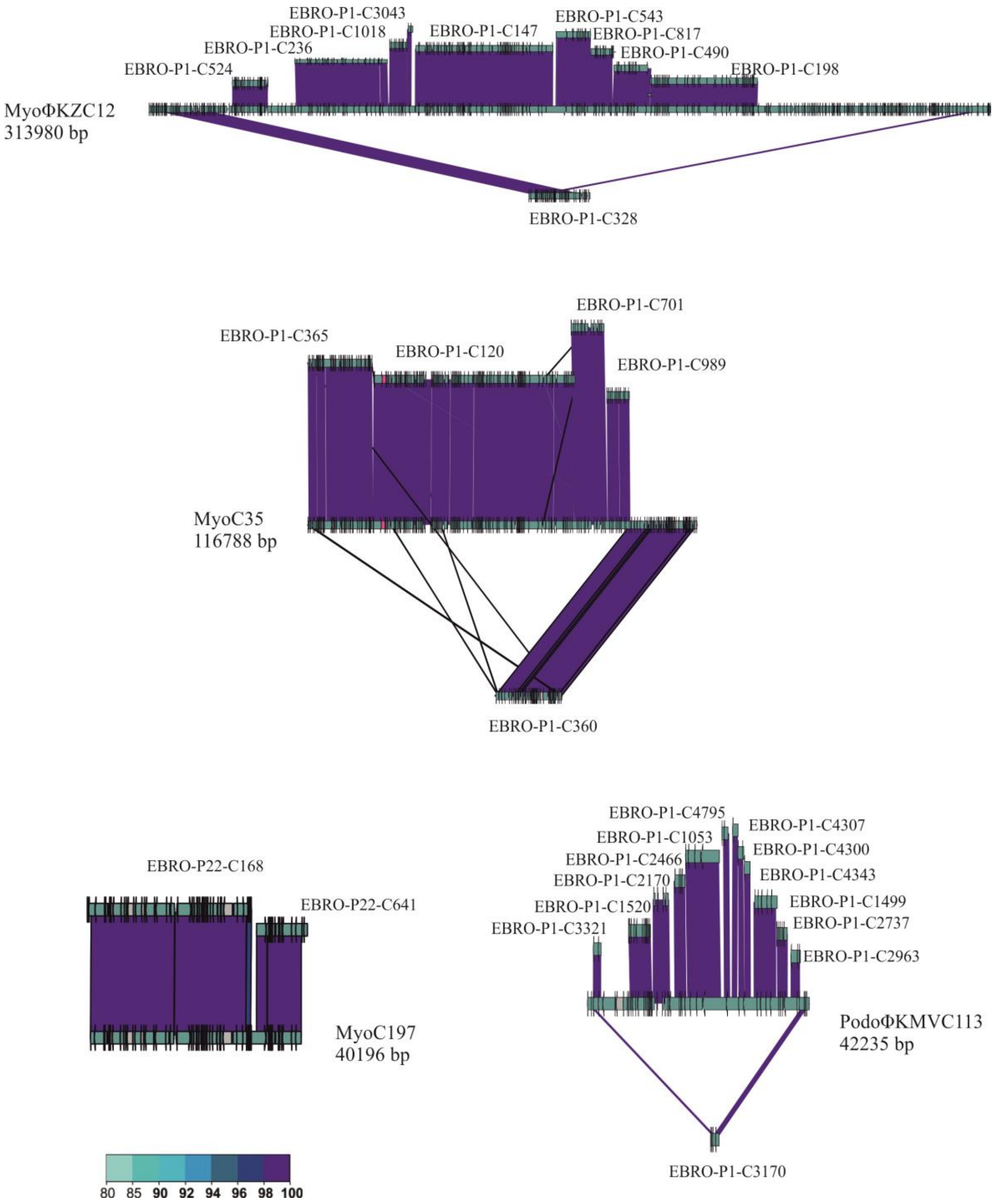

Supplementary Figure 2. Viral contigs alignment. All viral contigs larger than 10 Kb were aligned using TBLASTX. The identity of the alignment is represented by a colour code.

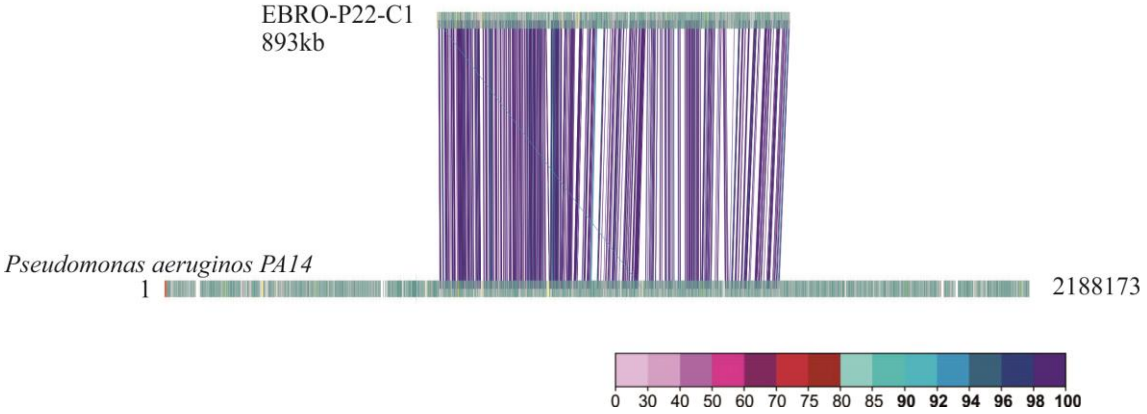

Supplementary Figure 3. Bacterial contig alignment. The largest bacterial contig assembled in the Ebro metagenome from 2013 aligned to a fragment of the chromosome one of *Pseudomonas aeruginosa PA14* using TBLASTX.

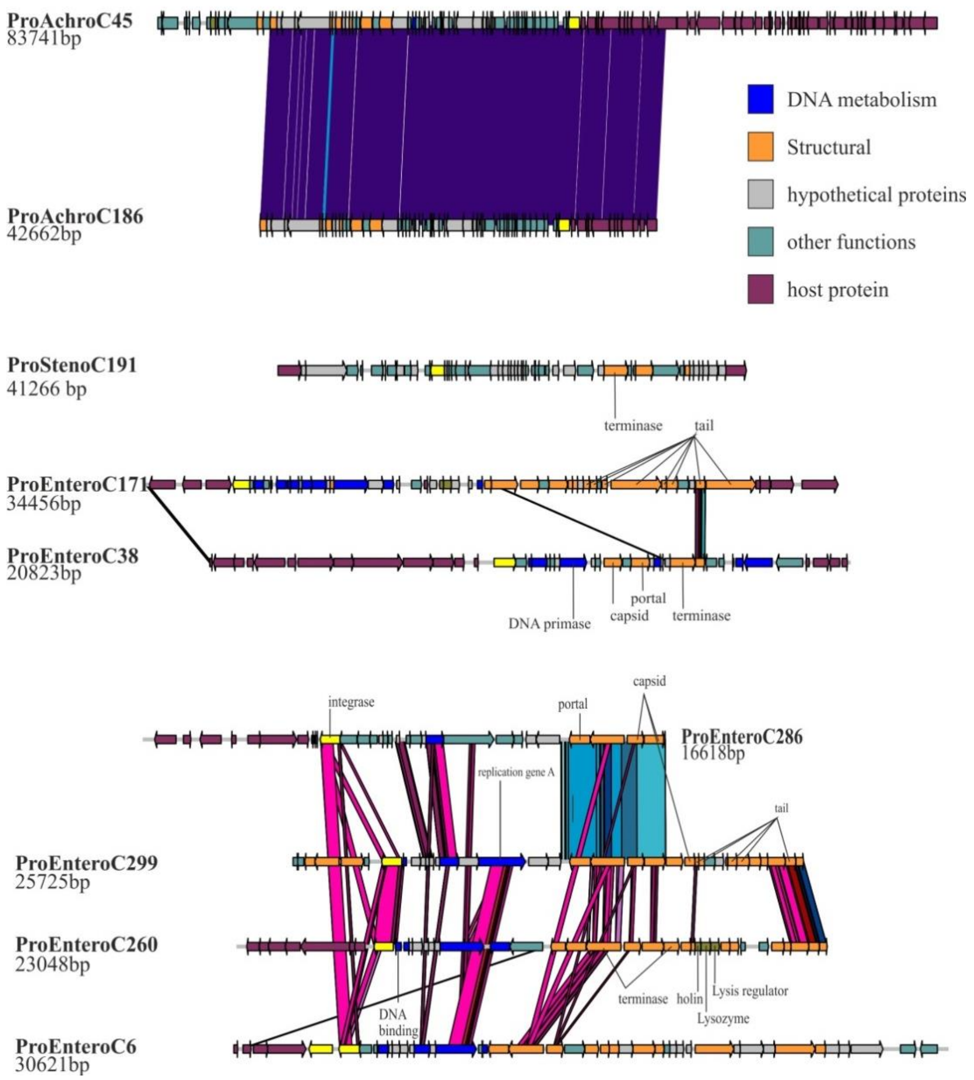

Supplementary Figure 4. Sequenced prophages present in Ebro Delta 2013. Prophages were annotated and compared between them.

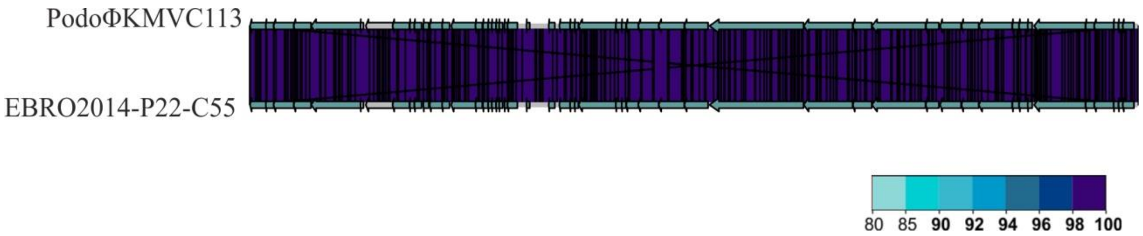

Supplementary Figure 5. PodoΦKMVC113 in Ebro Delta samples. Comparison of two complete genomes assembled in 2013 and 2014 (below).

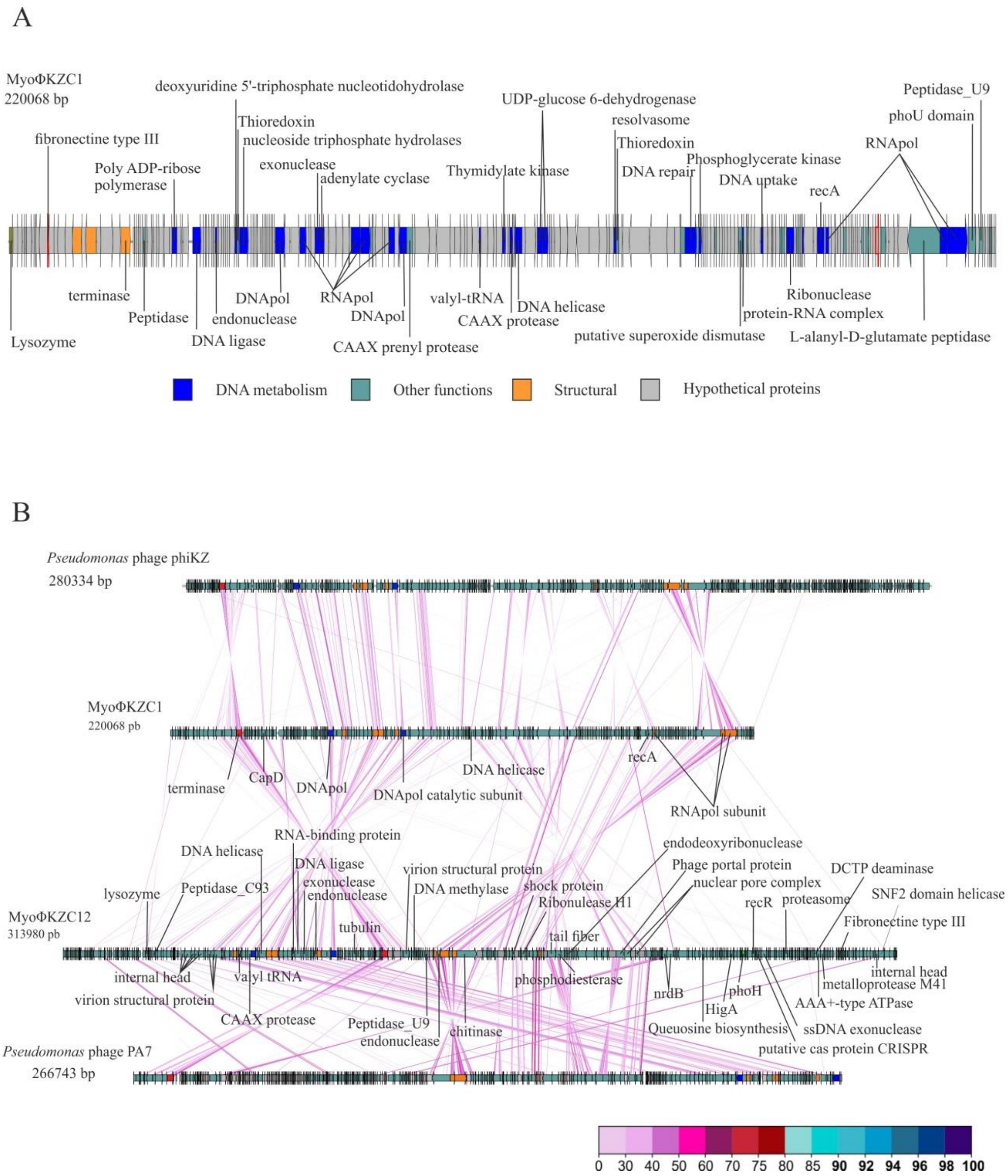

Supplementary Figure 6 Genome representation of MyoΦKZC1 and genome comparison of ΦKZ. A, The complete annotation of MyoΦKZC1 is represented by arrows and different colours were used to differentiate functions. Ig-domain is highlighted using a red fringe. B, TBLASTX comparisons were done between MyoΦKZC12, MyoΦKZC1, *Pseudomonas* phage phiKZ and PA7

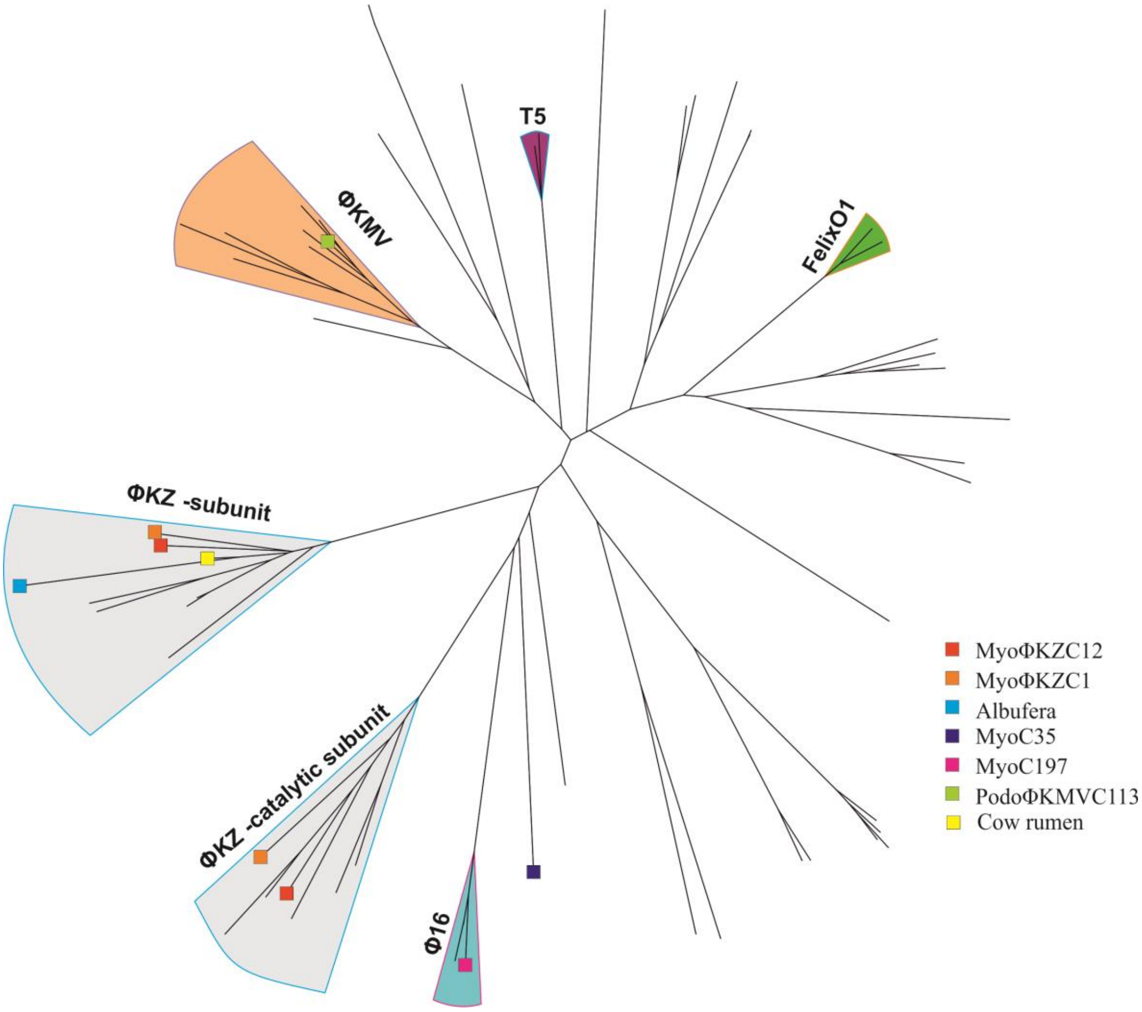

Supplementary Figure 7. DNA polymerase phylogeny. Maximum-likelihood tree build using DNApol genes identified in the sequenced genomes, Albufera and Cow rumen metagenomes are from Genbank database. Clearly clustered genus and new identified DNApol are labelled. The two ΦKZ subunits are labelled using the same colour.

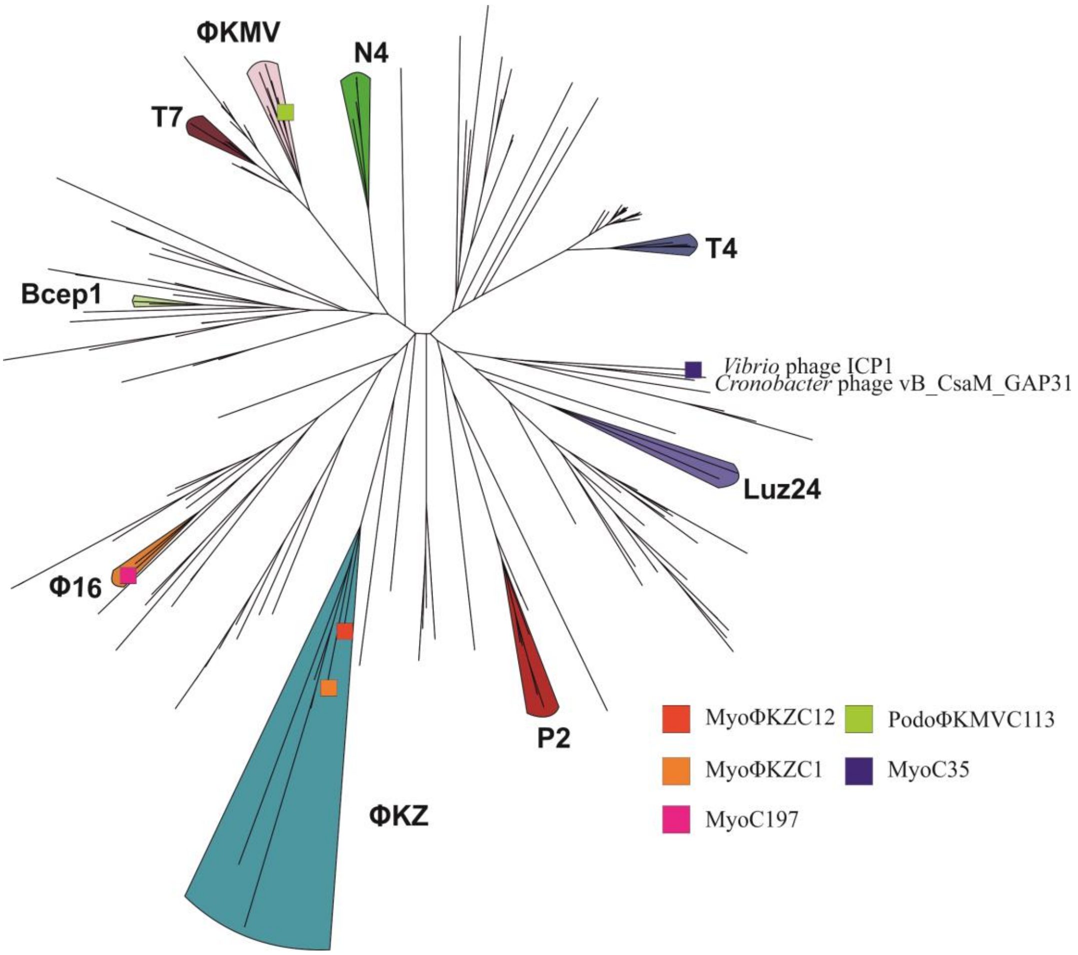

Supplementary Figure 8. Terminase phylogeny. Large terminase subunits recovered from all genomes and others selected from Genbank were used to construct a maximum-likelihood tree. Branches were labelled when genus were perfectly clustered in it. The branches corresponding to our data are coloured.

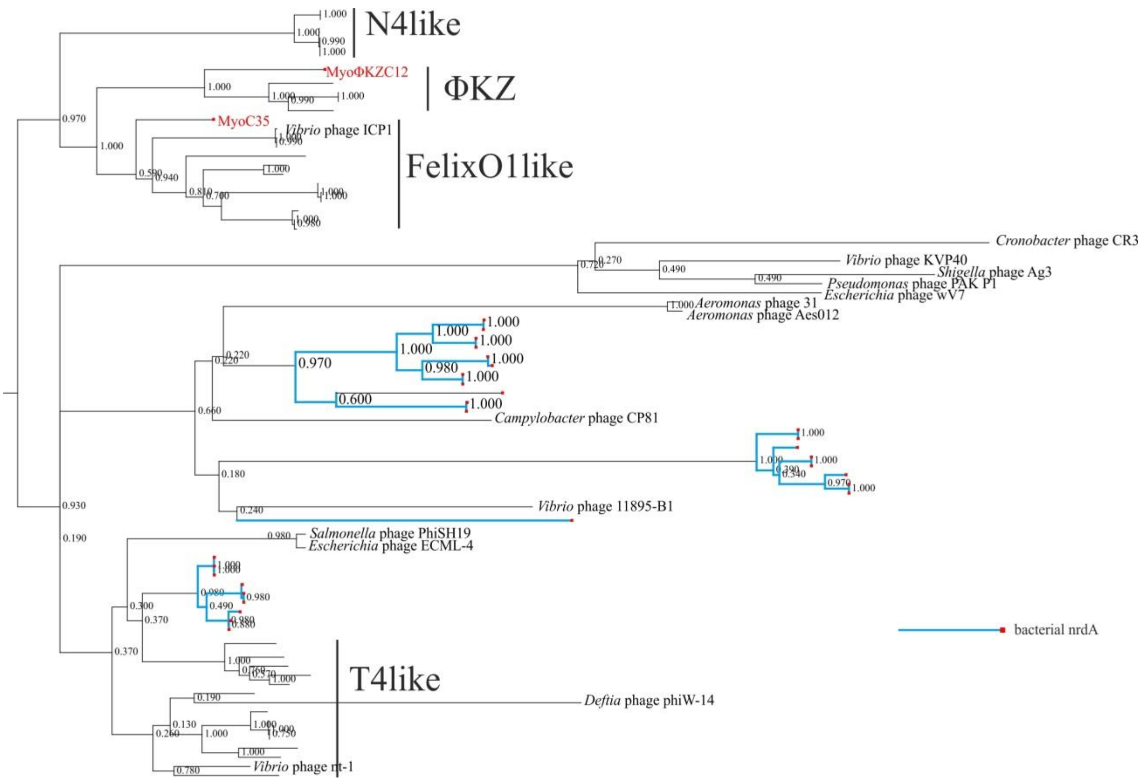

Supplementary Figure 9. Ribonucleoside diphosphate reductase alpha chain phylogeny. NrdA from the bacteriophages and the putative hosts were used for the analysis. Branches clustered in one genus were grouped using the genus name.

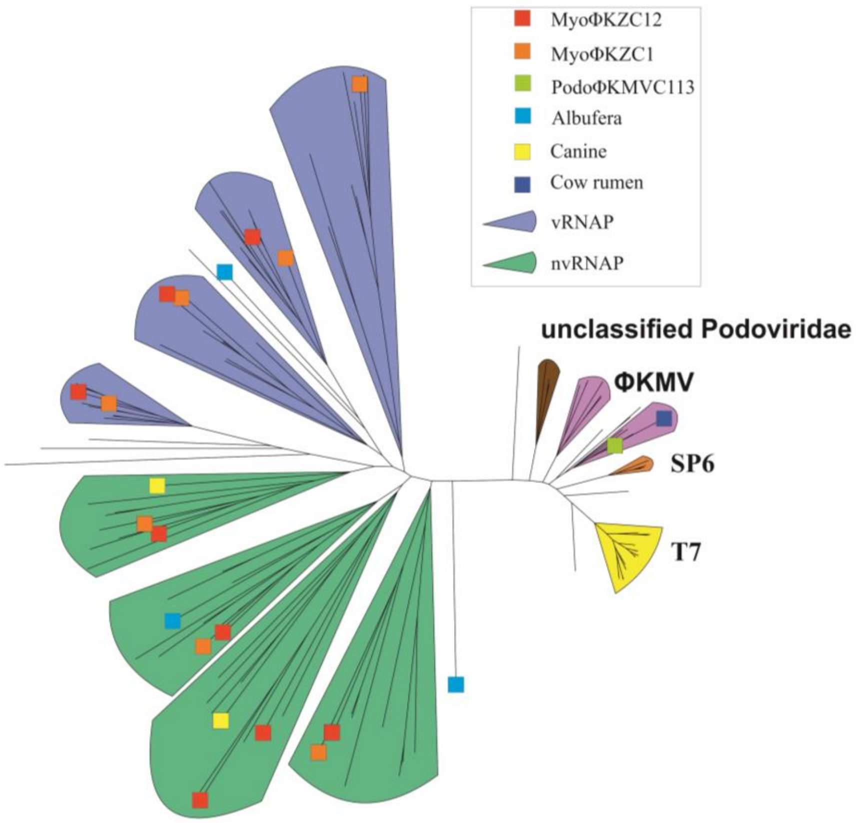

Supplementary Figure 10. RNA polymerase phylogeny. Maximum-likelihood tree using RNApol from Genbank database, MyoΦKZC12, MyoΦKZC1and PodoΦKMVC113 genomes and from Albufera, canine feces and cow rumen metagenome. Branches in which genera have been clustered separately were coloured differently and RNApol from ΦKZ members were coloured accordingly to the subunit. RNApol genes from metagenome of eels were marked with coloured boxes.

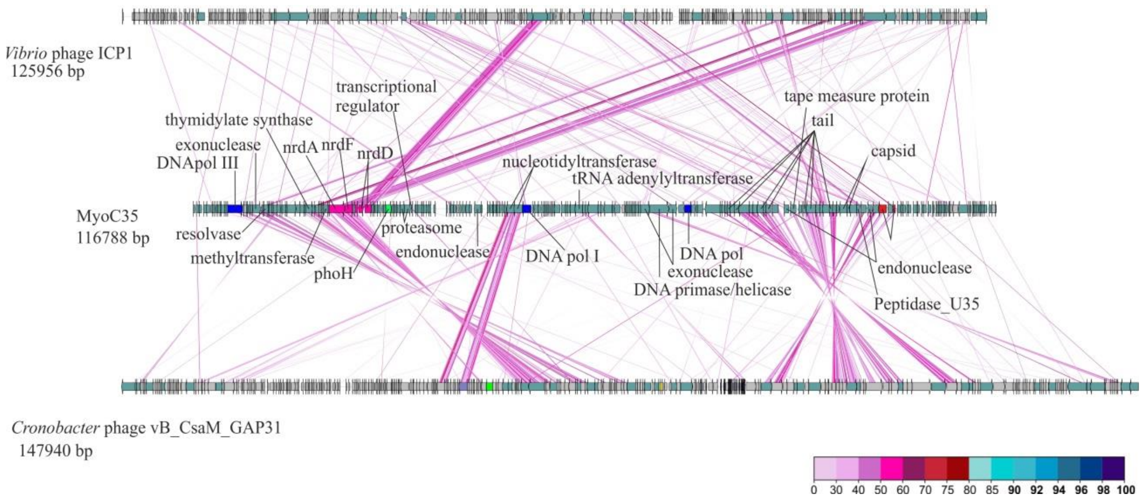

Supplementary Figure 11. Genome comparison of MyoC35 and the two most similar complete phages from databases. MyoC35 was compared against *Vibrio* phage ICP1 and *Cronobacter* phage vB_CsaM_GAP31 using TBLASTX.

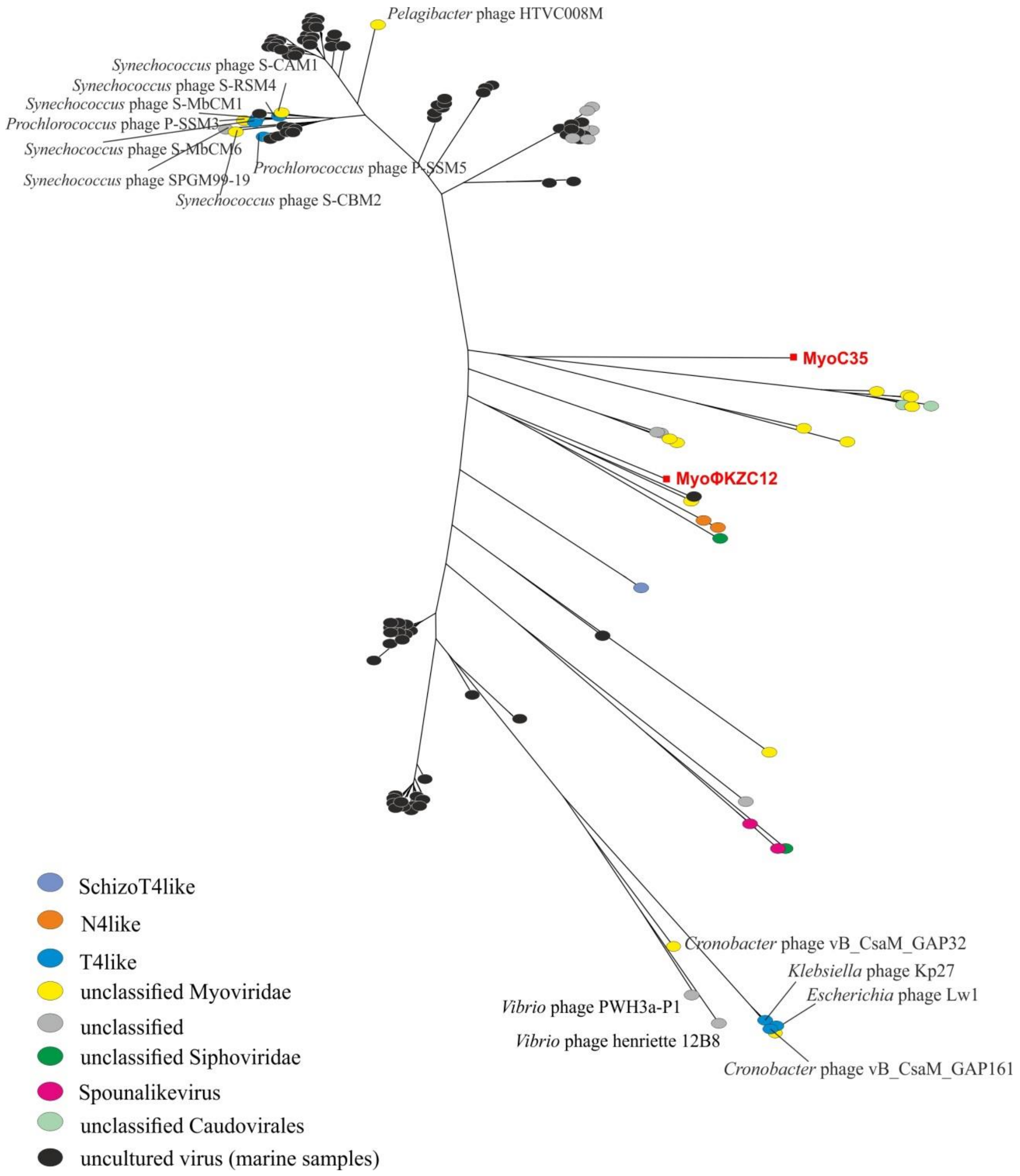

Supplementary Figure 12. Phylogenetic tree showing the clustering of *phoH* genes of virus isolated from marine samples and from cultured phages. *phoH* genes identified in our genomes are label in red. The taxonomy of the rest is labeled with different colours. The names of phage genomes which clustered with virus from marine samples were added.

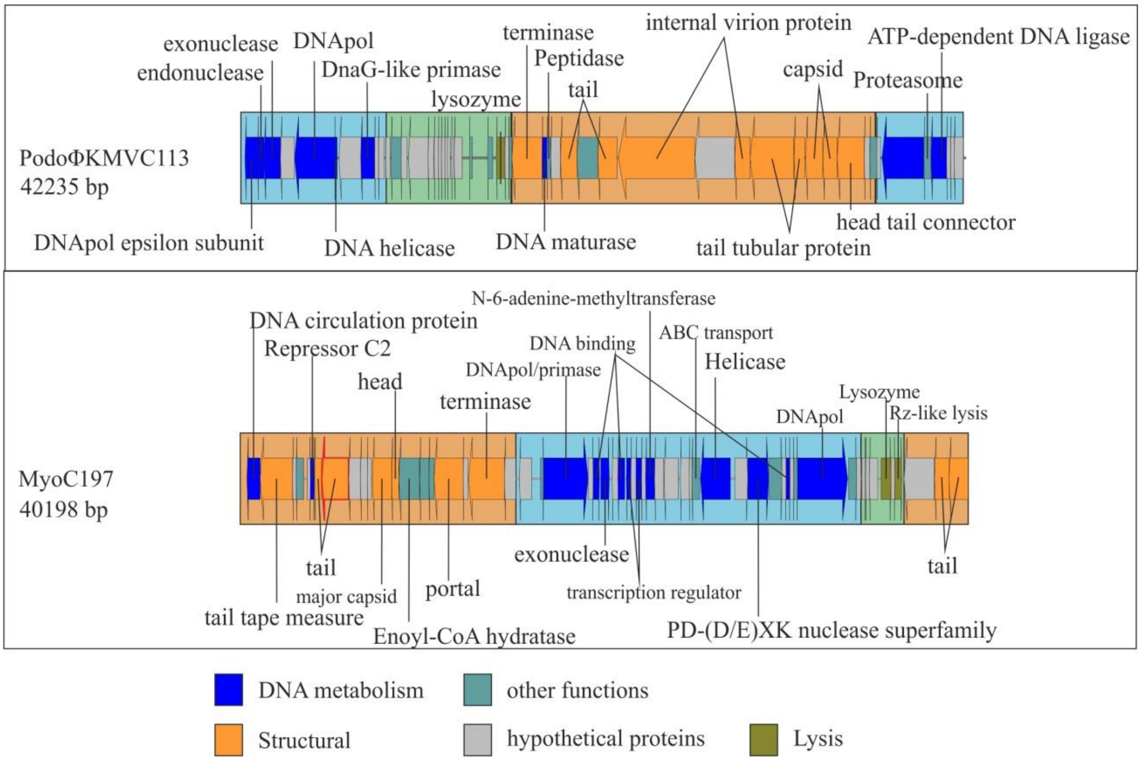

Supplementary Figure 13. Genome representation of PodoΦKMVC113 and MyoC197. Domains were boxed according to the function of the genes present in it: orange, blue and green indicating structural, DNA metabolism and lysis domains, respectively. Ig-like domains are highlighted using a red fringe.

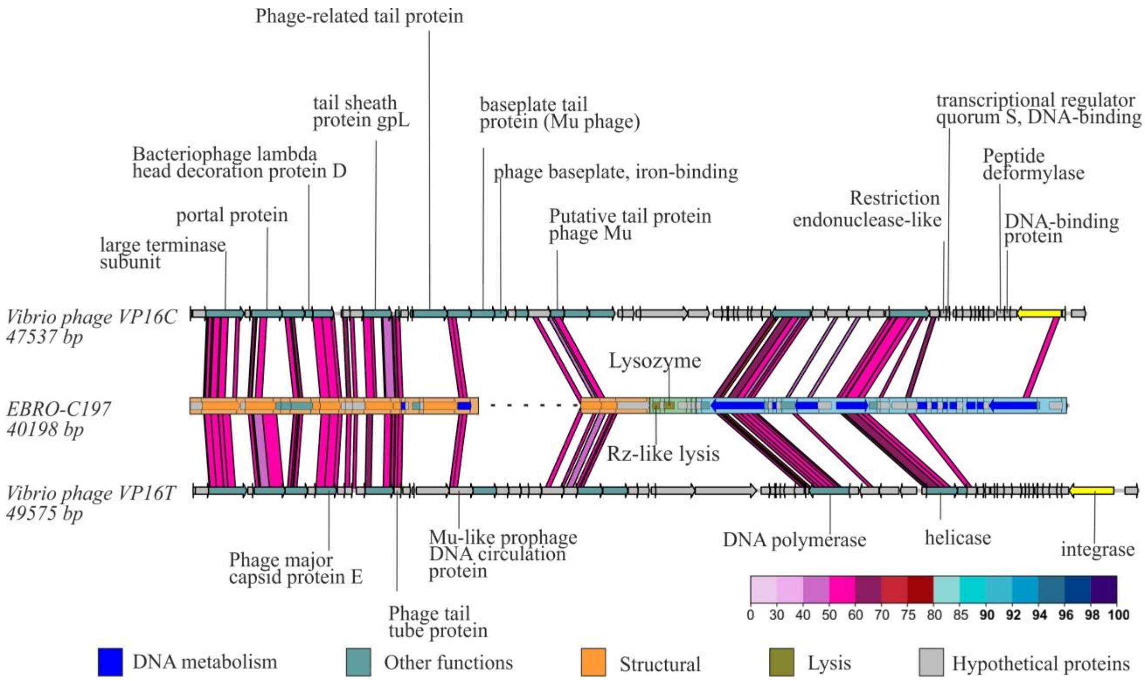

Supplementary Figure 14. ***Vibrio*** **phages comparison.** MyoC197 was compared against *Vibrio* phage VP16T and VP16C using TBLASTX. MyoC197 was oriented in the same sense as the *Vibrio* phages used for comparison. The annotation of MyoC197 was coloured according to function.
